# Recent advances in the management of knee osteoarthritis: a narrative review

**DOI:** 10.3389/fmed.2025.1523027

**Published:** 2025-01-21

**Authors:** Viktor Shtroblia, Pavlo Petakh, Iryna Kamyshna, Iryna Halabitska, Oleksandr Kamyshnyi

**Affiliations:** ^1^Department of General Surgery, Uzhhorod National University, Uzhhorod, Ukraine; ^2^Department of Biochemistry and Pharmacology, Uzhhorod National University, Uzhhorod, Ukraine; ^3^Department of Medical Rehabilitation, I. Horbachevsky Ternopil National Medical University, Ternopil, Ukraine; ^4^Department of Therapy and Family Medicine, I. Horbachevsky Ternopil National Medical University, Ternopil, Ukraine; ^5^Department of Microbiology, Virology, and Immunology, I. Horbachevsky Ternopil National Medical University, Ternopil, Ukraine

**Keywords:** osteoarthritis, NSAIDs, opioids, corticosteroids, pain, cytokines

## Abstract

Knee osteoarthritis (OA) is a common condition that causes pain and reduces the quality of life for many people. It also leads to high health and financial costs. Managing knee OA pain requires using different methods together for the best results. This review overviews current therapeutic options for knee OA pain, focusing on their efficacy, safety, and potential roles in clinical practice. Topical treatments, such as NSAIDs and capsaicin, offer significant pain relief with minimal systemic side effects and are suitable for initial therapy, together with nonpharmacologic interventions like exercise and, when relevant, weight loss. Oral analgesics, including acetaminophen and opioids, have limited efficacy and serious side effects, making them appropriate only for short-term or rescue therapy. Intra-articular injections, such as corticosteroids, hyaluronic acid, and platelet rich plasma, demonstrate varying levels of efficacy and safety. Nutritional supplements, including curcumin, *Boswellia serrata*, and glucosaminechondroitin combinations, offer modest benefits and are best used as adjuncts to standart treatment. Nonpharmacological treatments, such as transcutaneous electrical nerve stimulation (TENS), acupuncture, and local heat therapy, provide variable pain relief and should be customized based on individual patient responses. Targeted biologic agents, such as antibodies to TNF-α, IL-1, and NGF, hold promise for more precise pain relief; however, further research is required to establish their routine use. Treating knee OA pain should be personalized, combining several methods. Research must continue to improve treatments and make them safer.

## Introduction

Osteoarthritis (OA) is a disease of movable joints characterized by anatomical and physiological abnormalities, such as cartilage degradation, bone remodeling, osteophyte formation, joint inflammation, and loss of normal joint function. It begins with micro- and macro-damage to the joint, which activates maladaptive recovery reactions, leading to abnormal tissue metabolism (1).

Osteoarthritis is a major cause of chronic disability, primarily due to pain, the main symptom of the disease (2). Knee OA pain typically progresses from intermittent pain during exercise to more persistent chronic pain (3, 4). Symptoms such as pain and stiffness in OA contribute to functional limitations, with a well-documented relationship between pain severity and the degree of functional limitation (5). OA also imposes a serious burden on health and the economy (6, 7).

**Figure 1:**
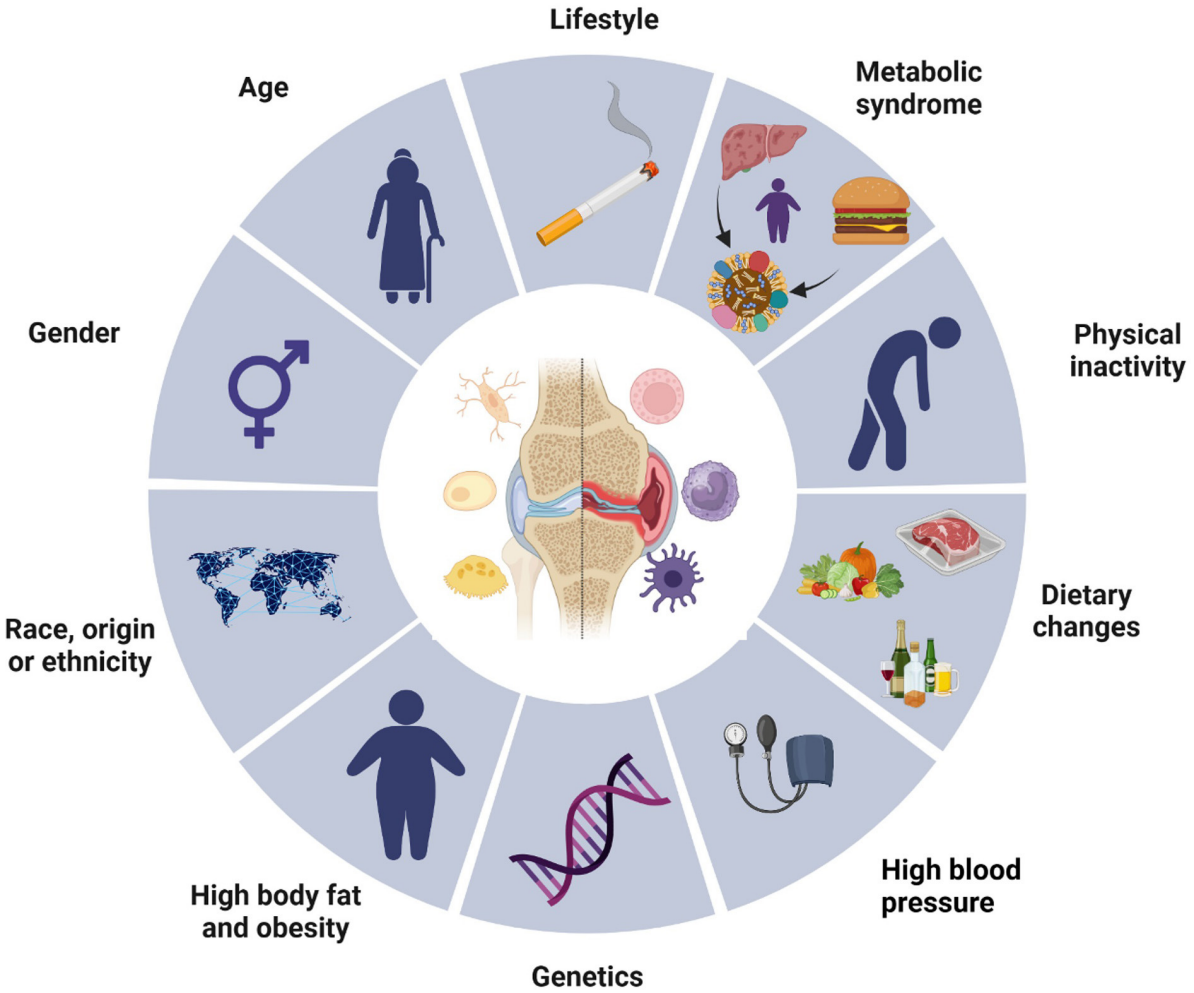
Osteoarthritis risk factors. This diagram illustrates various risk factors contributing to the development of osteoarthritis. The central image of a joint highlights the site of the condition, surrounded by multiple influencing elements segmented into lifestyle, biological, and genetic factors. Key components include age, gender, race or ethnicity, high body fat and obesity, genetic predispositions, and associated conditions such as metabolic syndrome and high blood pressure. Lifestyle choices, including physical inactivity, dietary habits, and behaviors like smoking, also play significant roles. Collectively, these elements emphasize the multifactorial nature of osteoarthritis, underscoring the complexity of its etiology.

Osteoarthritis is the most common musculoskeletal disease worldwide and represents a significant health and economic burden (8, 9). It is a major cause of chronic pain and disability due to reduced joint mobility and function and reduced quality of life (10, 11). Risk factors for osteoarthritis encompass genetic predispositions, lifestyle behaviors, biological factors such as age and gender, as well as metabolic conditions, including obesity and hypertension (Figure 1) (12, 13).

The knee is the most affected joint, accounting for ~85% of OA cases worldwide (14, 15). Knee joint osteoarthritis (OA) is a multifactorial disease characterized by various pathological changes, including cartilage degradation, osteophyte formation, remodeling of osteo cartilaginous units, and joint inflammation (16).

Various factors, including mechanical, inflammatory, aging, and metabolic disorders, contribute to the pathogenesis of OA (17–20). Dysbiotic alterations and stress are significant contributors to the progression of osteoarthritis and the exacerbation of pain syndromes (21–23). Therefore, it is essential to consider medications that can mitigate these factors (24–26). These factors ultimately lead to structural joint destruction, loss of synovial joint function, and long-term chronic pain (27–29). Patients with OA commonly experience stiffness, pain, and loss of function (30). The prevalence of OA increases with age: 13.9% of adults aged 25 years and older have clinical OA in at least one joint, whereas 33.6% of adults aged 65 years and older are affected. According to the Johnston County Osteoarthritis Project, the lifetime risk of developing symptomatic knee OA is ~45% (40% in men and 47% in women). This risk increases to 60.5% in obese individuals, which is approximately twice as high as the risk in those who are normal weight or underweight (31, 32). Pregnancy can exacerbate the progression of osteoarthritis due to increased weight and hormonal changes (33, 34). The coexistence of OA and endocrine disorders, especially those related to thyroid dysfunction, can complicate the clinical landscape, as metabolic alterations and hormonal imbalances linked to thyroid conditions may intensify inflammatory processes and promote the progression of osteoarthritis (35–37). Therefore, finding effective and safe treatments for OA is crucial in the clinic.

Pain in osteoarthritis arises from inflammatory, mechanical, and neuropathic mechanisms, requiring tailored management strategies. Mechanical pain is addressed through interventions that reduce joint stress, such as physical therapy, weight management, and the use of assistive devices, alongside systemic analgesics and intra-articular hyaluronic acid injections (38). Inflammatory pain is managed with NSAIDs and corticosteroid injections, while neuropathic pain responds to therapies like gabapentinoids, antidepressants, or radiofrequency ablation (39). Advanced regenerative treatments, such as platelet-rich plasma and stem cell therapy, show potential for addressing pain of mixed origin. Reducing pain remains the primary goal of osteoarthritis management, enhancing patient function and quality of life.

The aim of this review is to provide an in-depth evaluation of the current treatment strategies for knee osteoarthritis (OA), focusing on their comparative efficacy, safety profiles, and practical applicability in clinical settings. This review emphasizes recent advancements in topical and systemic pharmacological therapies, biologic agents, and emerging non-pharmacologic approaches, while identifying gaps in the evidence to guide future research.

## Topical treatment

### Topical NSAIDs

**Figure 2:**
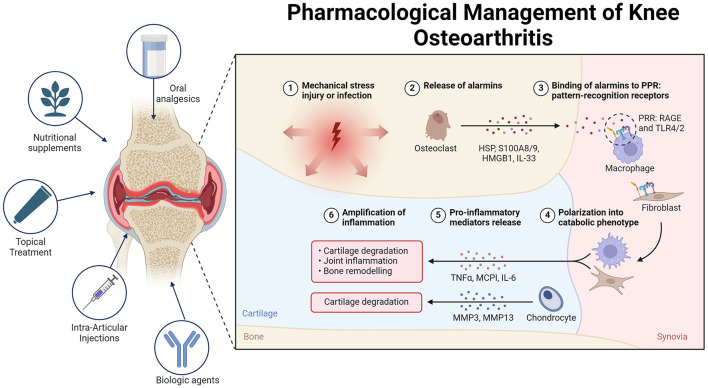
Overview of the pathogenesis and treatment of OA. In osteoarthritis (OA), alarmins are endogenous molecules released in response to various forms of damage. These molecules bind to pattern recognition receptors (PRRs) on different cells, triggering either an inflammatory or regenerative response. Alarmins can polarize cells such as macrophages and fibroblasts, leading to increased production of pro-inflammatory mediators and metalloproteases. This cascade of events contributes to cartilage destruction and joint damage, thus perpetuating inflammation and OA pathology. To manage OA pain, various treatment options are available. Topical treatments such as NSAIDs and capsaicin provide significant pain relief with minimal systemic side effects, making them suitable for initial therapy. Oral analgesics, such as acetaminophen and opioids, offer limited efficacy and have notable side effects, making them suitable only for short-term or rescue therapy. Intra-articular injections, including corticosteroids, hyaluronic acid, and platelet-rich plasma, show varying degrees of efficacy. Mesenchymal stem cells (MSCs) hold promise for future treatment pending further research. Nutritional supplements such as curcumin, *Boswellia serrata*, and glucosamine-chondroitin combinations present modest benefits and are best used as adjuncts. Non-pharmacological treatments, including transcutaneous electrical nerve stimulation (TENS), acupuncture, and local heat therapy, provide variable pain relief and should be considered on the basis of individual patient response. Biological agents that target cytokines such as TNF-α, IL-1, and NGF show promise, although additional research is necessary to establish their routine use.

A network meta-analysis revealed that topical and oral NSAIDs offer similar improvements in function and are more effective than paracetamol for treating knee osteoarthritis (OA). Data from 122 randomized controlled trials indicate that topical NSAIDs have a lower risk of gastrointestinal side effects than both paracetamol and oral NSAIDs do (40). Furthermore, real-world data suggest that topical NSAIDs have better overall safety than oral NSAIDs do. They also present lower risks of all-cause mortality, cardiovascular disease, and gastrointestinal bleeding than paracetamol in real-world settings (41) (Figure 2).

A Cochrane review revealed that ~60% of patients experienced at least a 50% reduction in pain with topical NSAIDs, comparable to the relief provided by oral NSAIDs and slightly better than that achieved with a topical placebo (42). Compared with oral formulations, topical NSAIDs have a much lower risk of gastrointestinal, kidney, and cardiovascular toxicity due to reduced systemic absorption [e.g., topical diclofenac is 5- to 17-fold less absorbed than the oral version; (43–45)].

Guidelines consistently endorse the use of topical NSAID therapy. The AAOS supports the use of topical NSAIDs for symptomatic treatment of knee OA (46). The OARSI guidelines recommend topical NSAIDs as a first-line treatment for knee OA pain relief. In contrast, the ACR/AF strongly advocates their use, suggesting that they be prioritized over oral NSAIDs (47). Similarly, the ESCEO guidelines advise the use of topical NSAIDs before oral NSAIDs when optimal pain relief is not achieved with first-line SYSADOA and acetaminophen (48) (Table 1).

**Table 1:** Society guidelines for oral and topical pharmacological agents in osteoarthritis.

**Society guideline**	**Year**	**Oral NSAIDs**	**Topical NSAIDs**	**Oral opioids**	**Acetaminophen**	**SYSADOA**
NICE	2022	If topical medicines are ineffective or unsuitable	First-line therapy	Do not offer	Do not routinely offer	No strong evidence of benefit
OARSI	2019	Conditionally recommended	Strongly recommended	Strongly not recommended	Conditionally not recommended	Not included
Choosing wisely Canada	2023	Strongly recommended	Strongly recommended	Do not recommend due to significant risk of side effects	Not included	Not included
AAOS	2022	Appropriate	Appropriate	Prescription should be monitored; intermittent or low dose in conjunction with other therapies	Appropriate	Not included
Royal Australian College of General Practitioners (RACGP)	2018	Conditionally recommended	Conditionally (neutral) recommended	Strongly against recommendation	Conditionally (neutral) recommended	Conditionally against recommendation

### Topical capsaicin

Capsaicin, a compound extracted from hot chili peppers, may help relieve pain by downregulating TRPV1 receptor activity on nociceptive sensory neurons and depleting substance P levels. With the ongoing use of capsaicin, nociceptive fibers become desensitized, reducing pain signal transmission. However, the role of substance P depletion in the pain-relieving effects of capsaicin has been called into question (49).

We found three systematic reviews examining the efficacy of capsaicin for osteoarthritis (OA) pain relief. In the first review by Cameron et al., five randomized controlled trials (RCTs) involving 456 participants were analyzed (50). The intervention involved applying topical capsaicin (0.025% or 0.075%) four times daily, compared with a placebo, over a follow-up period of 3–4 weeks. The primary endpoint was pain assessment, which was mostly measured by a visual analog scale (VAS). The study concluded that topical capsaicin significantly reduced OA pain in the hand, knee, or multiple joints and was superior to placebo. However, the blinding was compromised because of the local burning sensation associated with capsaicin.

In the second review by De Silva et al., five RCTs involving 427 participants were included (51). The intervention included topical capsaicin (0.015%, 0.025%, or 0.075%), which was applied once or four times daily, rather than a placebo, over a 4–12 weeks follow-up period. The primary endpoint was also pain assessment. The results indicated that topical capsaicin was significantly more effective than placebo in relieving hand and knee OA pain. Redness and local burning sensations were the capsaicin group's most frequently reported side effects. This review was also assigned a level of evidence of 2.

In the third review by Laslett and Jones, five RCTs and one case-crossover trial involving 1162 participants were analyzed (52). The intervention involved topical capsaicin (0.025% or 0.075%) applied four times daily, compared with a placebo, over a 4–12 weeks follow-up period. Pain assessment, primarily measured by the VAS, was the endpoint. The review revealed that topical capsaicin had moderate efficacy in reducing pain intensity in OA of the hand, knee, or several joints compared with placebo. Mild localized burning was the most frequently reported adverse event, but its incidence decreased with continued use.

In a 12 week randomized, multicenter trial involving 113 patients, participants were treated with either 0.025% capsaicin cream or a placebo applied four times daily. The findings indicated that capsaicin led to more significant pain relief over the 4 to 12 week period. Furthermore, 81% of patients in the capsaicin group reported improvement according to clinicians' global evaluations, whereas 54% of patients in the placebo group reported improvement (53).

## Oral analgesics

### Acetaminophen

Acetaminophen is frequently used as a first-line analgesic for various painful conditions. However, a meta-analysis of 10 trials involving 3,541 patients revealed high-quality evidence indicating that acetaminophen offers only small, non-clinically meaningful benefits for short-term pain relief (54). This conclusion was further supported by a network meta-analysis comparing different analgesics for OA pain, which revealed that acetaminophen was no better than placebo, regardless of the dose (showing a 4 mm difference on a 0–100 mm visual analog scale [VAS]) (55). The risk of harm from acetaminophen typically increases with higher doses but can also occur at therapeutic doses, including risks of gastrointestinal bleeding, liver toxicity, kidney failure, and cardiovascular disease (56, 57).

The American College of Rheumatology/Arthritis Foundation (ACR/AF) issued a conditional recommendation for using acetaminophen due to its small effect size when used as monotherapy. It may be suitable for short-term or periodic use in patients who cannot take other analgesics (47). The European Society for Clinical and Economic Aspects of Osteoporosis, Osteoarthritis, and Musculoskeletal Diseases (ESCEO) 2019 guidelines also provide a conditional recommendation for acetaminophen, suggesting its use only for short-term rescue analgesia in combination with long-term chondroitin sulfate or glucosamine (48). The American Academy of Orthopaedic Surgeons (AAOS) did not make a recommendation for or against acetaminophen use (46). Despite its widespread use, acetaminophen should be prescribed with caution because of its known side effects. In some patients, higher doses or prolonged use can lead to hepatotoxicity (58).

### Non-steroidal anti-inflammatory drugs

Non-steroidal anti-inflammatory drugs (NSAIDs) are commonly employed for pain management in osteoarthritis because they inhibit the cyclooxygenase (COX) enzyme, resulting in reduced production of prostaglandins, which play a critical role in mediating inflammation and pain (59–61). This category includes both conventional NSAIDs, such as ibuprofen and diclofenac, and selective COX-2 inhibitors, such as celecoxib, known for a lower incidence of gastrointestinal side effects (62–64). International guidelines, including those from the Osteoarthritis Research Society International (OARSI) and the American College of Rheumatology (ACR), advocate for the use of NSAIDs as first-line treatment for pain relief in osteoarthritis (47, 65). Nonetheless, long-term use necessitates careful monitoring due to potential risks, notably gastrointestinal, cardiovascular, and renal adverse effects (66–69).

### Opioids

Due to their relatively high incidence of side effects, including drowsiness, dizziness, and nausea, as well as the potential for harm with long-term use, opioids are typically prescribed for osteoarthritis (OA) only when other analgesics have proven ineffective or are contraindicated (70). They are also considered for patients who are not candidates for joint replacement. Studies on knee OA have shown that opioids reduce pain to a similar degree as NSAIDs. A meta-analysis indicated a modest effect size (standardized mean difference [SMD] −0.28, 95% CI −0.35 to 0.20) for non-tramadol opioids, translating to a 0.7 cm difference on a 0–10 cm visual analogue scale (VAS) compared with placebo (71). Improvements in knee function were also limited, and the daily morphine equivalent dose did not impact functional benefits. Patients on opioids were more likely to discontinue treatment because of adverse events and experienced more side effects (6.5% vs. 1.7% and 22% vs. 15%, respectively; 71). Moreover, a randomized trial with 240 patients suffering from chronic back pain or hip or knee OA pain reported no difference in pain-related function after 12 months of treatment with non-opioid or opioid medications (72).

Moreover, less-potent opioids do not seem to offer significant advantages over non-opioid medications. A network meta-analysis did not reveal differences in efficacy between potent opioids (such as hydromorphone and oxycodone), a less-potent opioid (tramadol), and NSAIDs in trials lasting at least 8 weeks (73). A meta-analysis of six trials involving 3,611 patients with knee or hip OA revealed that tramadol provided modest pain relief compared with placebo, with only the high dose (300 mg/day) showing improvements in the functional subscale of the Western Ontario and McMaster Universities Osteoarthritis Index (WOMAC) compared with the placebo [SMD −0.24, 95% CI −0.47 to 0.03; (74)].

In addition to the known risks associated with opioid use, tramadol may be linked to increased mortality in OA patients. A propensity score-matched study of 88,902 OA patients revealed that patients prescribed tramadol had a higher mortality rate over a 1 year follow-up than did those taking commonly prescribed NSAIDs such as naproxen (hazard ratio 1.71 [95% CI 1.41–2.07]) (75).

A systematic review and meta-analysis examining opioid use for OA pain revealed poor tolerability and minimal clinical benefit of opioids in controlled studies lasting between 4 and 24 weeks (76). Similarly, a recent meta-analysis by Osani et al. revealed that opioids provided only minor improvements in pain and function compared with placebo over 2–12 weeks of treatment, with no significant improvement in patients' quality of life. A meta-analysis revealed that more potent opioids, such as morphine and oxycodone, offered less favorable clinical outcomes than weaker or intermediate opioids, such as codeine and tramadol, and were associated with a greater risk of adverse effects (77).

## Intra-articular injections

### Corticosteroid injections

Injected corticosteroids target specific areas, such as inflammation or pain from tendinitis or osteoarthritic joints. A Cochrane review on intra-articular corticosteroid injections revealed that these treatments could provide moderate pain relief and slight improvements in physical function. However, the side-effect profile of intra-articular corticosteroids was comparable to that of a placebo. The evidence quality was deemed very low because of significant inconsistencies among the study results and the reliance on numerous small, low-quality studies (78).

Despite their common use in clinical practice and short-term effectiveness for joint pain relief, recent studies indicate that intra-articular glucocorticoid injections are less effective than physical therapy in managing symptoms 1 year after administration (79).

### Hyaluronans

Hyaluronic acid (HA) is a glycosaminoglycan with various therapeutic effects when injected intra-articularly, including anti-inflammatory, mechanical, and analgesic benefits, as well as a positive impact on proteoglycan and glycosaminoglycan synthesis (80). A systematic review by Altman et al. revealed that repeated HA injections could maintain or improve knee pain without increasing safety risks, highlighting the advantage of the safety of repeated HA injections (81). Recent improvements in HA products have led to the development of high-molecular-weight hyaluronic acid (HMWHA), which is thought to be more effective than low-molecular-weight hyaluronic acid [LMWHA; (80)]. This finding was supported by a systematic review showing that HMWHA had a more significant impact than non-selective NSAIDs and selective COX-2 inhibitors for treating knee osteoarthritis [OA; (82)]. Additionally, a systematic review and meta-analysis by Miller et al. revealed that intra-articular HA injections resulted in statistically significant, although not clinically important, improvements in pain and knee function, with fewer side effects than orally administered NSAIDs did (83).

### Platelet-rich plasma

Studies generally agree on the short- and medium-term analgesic effects of platelet-rich plasma (PRP) in patients with knee osteoarthritis (OA). However, drawing definitive conclusions about its clinical efficacy is challenging owing to variations in PRP preparation and application methods (84, 85). A meta-analysis of 40 trials involving 3,035 knee OA patients revealed no significant improvement in pain or function with PRP compared with hyaluronic acid, intra-articular steroids, or saline (86). Additionally, a randomized trial of 288 patients included in the meta-analysis revealed that intra-articular PRP injections did not provide benefits in terms of pain relief or structural changes compared with a saline placebo (87).

### Mesenchymal stem cells (MSCs)

Autologous bone marrow-derived mesenchymal stem cells (BM-MSCs) and adipose-derived MSCs (AD-MSCs), also known as the adipose-derived stromal vascular fraction (AD-SVF), are commonly used for treating knee osteoarthritis (OA). These cells can be either cultured before application or used directly after isolation. Other cell sources, such as synovial MSCs or allogeneic placental tissue, still require more research before they become routine in clinical practice (88).

During the progression of OA, MSCs applied directly into the joint tend to accumulate in both the joint and nearby bone marrow lesions, suggesting that they play a role in the response to joint injury. However, the precise mechanism behind their effectiveness in OA is not fully understood (89). Despite this, MSCs are increasingly employed in clinical settings, with reports indicating benefits in symptom relief and joint function (90–92).

One meta-analysis that included five randomized controlled trials (RCTs), four involving BM-MSCs and one involving AD-SVF, reported a significant reduction in pain intensity, as measured by the visual analog scale (VAS) and the Lysholm scale. However, no difference was noted in the Western Ontario and McMaster Universities Osteoarthritis Index (WOMAC) scores. The functional outcomes significantly improved, with a standard mean difference of 0.53%, although no notable difference in cartilage repair on MRI was observed (93).

Another meta-analysis reviewed RCTs of culture-expanded MSCs for OA treatment, including six studies (four with BM-MSCs, one with AD-MSCs, and one with placenta-derived MSCs) and 203 patients. This analysis revealed a statistically significant reduction in pain symptoms measured by both the VAS and WOMAC. Still, it revealed no significant difference in cartilage repair based on MRI or the whole-organ magnetic resonance score [WORMS; (94)].

Further analysis by Ma et al., which included 10 RCTs (four with BM-MSCs, three with AD-MSCs, one with adipose-derived mesenchymal progenitor cells [AD-MPCs], one with umbilical cord MSCs, and one with placenta-derived MSCs), revealed a significant reduction in pain, as measured by the VAS and WOMAC, along with improved stiffness, functionality, and total WOMAC scores. This study also reported increased cartilage volume among MSC-treated patients, although no significant difference was found in WORMS scores (95).

A comprehensive meta-analysis of 19 studies (15 RCTs, two retrospective studies, and two cohort studies), including nine with AD-MSCs, five with BM-MSCs, and others with peripheral blood stem cells or fetal MSCs, revealed significant pain relief at 12 months and improvements in the KOOS and WOMAC scores at 6 months. No side effects were reported from intra-articular MSC therapy (96).

In contrast, a systematic review and meta-analysis by Maheshwer et al. involving 25 studies reported no significant improvement in pain but reported functional and cartilage volume improvements, with standardized mean differences of 0.66 and 0.84, respectively (97).

### Botulinum toxin

Botulinum toxin (BTX), a complex multi-molecular substance synthesized by various strains of the anaerobic bacterium *Clostridium botulinum*, has shown potential therapeutic effects in managing OA (98, 99). Administering Botulinum neurotoxin type A directly into the joint may suppress the release of inflammatory mediators and neuropeptides from nociceptors, leading to reduced pain and neurogenic inflammation associated with OA (100). Additionally, BTX may exhibit anti-nociceptive properties by down-regulating voltage-gated sodium channels, as demonstrated in a rat model of trigeminal neuralgia, or by diminishing the peripheral release of neurotransmitters such as substance P and CGRP, along with the pro-inflammatory cytokine IL-1β (101–103). Furthermore, BTX inhibits the fusion of intracellular vesicles with nerve membranes, disrupting the release of neurogenic inflammatory mediators (104, 105). Clinical studies have noted that a single intra-articular injection of BTX can alleviate symptoms in some patients with chronic, refractory pain due to knee OA, while others show no significant benefit, hinting at the possibility of distinct patient subgroups (106). This evidence supports the off-label use of botulinum toxin as a novel therapeutic strategy for KOA management in orthopedic practice (107).

## Pleiotropic effects of medications in osteoarthritis therapy

### Metformin

Metformin, a commonly prescribed medication for the management of type 2 diabetes, has attracted increasing interest in recent years for its potential uses beyond glycemic control. Initially designed to enhance insulin sensitivity and regulate hyperglycemia, metformin exhibits a range of pleiotropic effects that may be particularly advantageous in addressing various inflammatory and metabolic disorders, including OA (108–110). Research indicates that the aanti-inflammatory properties of metformin may significantly contribute to the reduction of joint degradation in OA patients (111, 112). By influencing inflammatory pathways and cellular stress responses, metformin may aid in preserving cartilage and soft tissues within the joints, which are often vulnerable to damage caused by inflammation (113, 114). Such mechanisms could lead to improvements in physical function and reductions in pain levels among OA patients, positioning metformin as a promising adjunctive therapy for those suffering from joint-related conditions (115, 116).

Furthermore, metformin's pleiotropic effects extend to its potential application in managing COVID-19, where it may help alleviate the severe inflammatory responses associated with the virus, particularly in high-risk groups such as individuals with obesity and diabetes (117–119). By modulating immune responses and decreasing the secretion of pro-inflammatory cytokines, metformin could reduce the likelihood of complications related to COVID-19, thereby underscoring its significance as a versatile therapeutic agent (120–122).

Metformin represents a promising adjunctive therapy for osteoarthritis, owing to its anti-inflammatory effects and ability to maintain joint integrity (123, 124). Its pleiotropic effects not only enhance physical function and alleviate pain in OA patients but also suggest possible applications in the treatment of other conditions, including COVID-19 (125–128). This highlights the broader therapeutic implications of this extensively utilized medication (129, 130). The dual benefits of metformin in managing osteoarthritis, along with its potential role in addressing COVID-19, emphasize its relevance as a multifaceted treatment option for patients with comorbid conditions, ultimately contributing to enhanced overall health outcomes (131–133).

### Statins

Statins, a class of medications primarily prescribed for lowering cholesterol levels and managing cardiovascular health, have garnered attention in recent years for their potential benefits beyond lipid regulation (134, 135). Research indicates that statins possess notable anti-inflammatory properties that may play a crucial role in the management of OA (136, 137). These medications have been found to reduce systemic inflammation, which is a significant contributor to the pathophysiology of OA (138, 139).

Statins may help slow the progression of OA by mitigating these inflammatory processes (140, 141). By inhibiting the production of pro-inflammatory cytokines and promoting the expression of anti-inflammatory mediators, statins can help create a more favorable environment within the joint, potentially preserving cartilage and soft tissue integrity (142, 143).

In addition to their direct anti-inflammatory effects, statins may enhance the synthesis of cartilage components such as proteoglycans and collagen (144, 145). This is particularly important because the degradation of these components is a hallmark of OA progression (146–148). By supporting cartilage maintenance and repair, statins could improve joint function and reduce symptoms for individuals with osteoarthritis (149, 150).

Moreover, the pleiotropic effects of statins extend beyond inflammation and cartilage preservation (151, 152). Evidence suggests that statins may protect bone health, further supporting joint integrity in OA patients (141, 153). By influencing bone remodeling and reducing the risk of osteoporotic changes, statins may help mitigate one of the risk factors associated with OA progression (154–156).

### Angiotensin-converting enzyme inhibitors and angiotensin II receptor blockers

Angiotensin-converting enzyme (ACE) inhibitors and angiotensin II receptor blockers (ARBs), primarily known for managing hypertension and heart failure, have shown promising potential in addressing OA due to their ability to modulate the renin-angiotensin-aldosterone system [RAAS; (157–160)]. By inhibiting the action of angiotensin II, these medications can reduce inflammation and oxidative stress, significantly contributing to joint degeneration in OA (161, 162). Research indicates that ACE inhibitors and ARBs may decrease levels of pro-inflammatory cytokines and oxidative stress markers in joint tissues, thereby alleviating inflammation and potentially slowing the progression of the disease (163, 164).

Furthermore, the protective effects of ACE inhibitors and ARBs may extend to the preservation of cartilage and synovial fluid, which are vital for joint integrity and function. By mitigating harmful inflammatory mediators, these medications may help maintain cartilage structure and improve the lubrication of joints, leading to enhanced mobility and reduced pain for patients with OA (165–167). This highlights the importance of a holistic treatment approach, as patients with OA often have comorbidities such as hypertension and obesity (168, 169). By integrating ACE inhibitors and ARBs into the management strategy for OA, healthcare providers can address joint health and overall cardiovascular risk, ultimately improving patients' quality of life (170).

## Nutritional supplements

### Curcumin (turmeric)

Interest in curcumin is due primarily to its potential anti-inflammatory and analgesic effects, although evidence remains limited (171). Randomized trials and meta-analyses have investigated its efficacy (172, 173). For example, a study involving 70 adults with knee osteoarthritis (OA) and ultrasound-confirmed effusion synovitis revealed that 1,000 mg of Curcuma longa daily provided more significant pain relief over 12 weeks than a placebo. However, the clinical significance of these findings is questionable, as the observed improvements were more than the minimal clinically significant difference. Additionally, measures of effusion-synovitis volume on MRI were similar between the curcumin and placebo groups, with comparable adverse events reported. More extensive trials are needed to establish the clinical relevance of curcumin in OA treatment. Curcumin is known for its poor gastrointestinal absorption, so supplements designed to increase its bioavailability, such as those combined with piperine or BioPerine, are typically preferred. Reports of liver injury associated with high-dose curcumin supplements are rare (174).

###  *Boswellia serrata*

*Boswellia serrata*, also known as Indian frankincense, has been used for centuries because of its anti-inflammatory and antimicrobial properties (175). A meta-analysis of seven randomized trials comparing *Boswellia serrata* extract with a placebo for OA suggested potential benefits in reducing pain and stiffness and improving function. However, the quality of the trials was low, with several studies having an unclear risk of bias. While Boswellia treatment was generally well tolerated, three included studies did not report adverse events (176).

### Glucosamine and chondroitin

The effectiveness of glucosamine and chondroitin in treating knee OA has been inconsistent (177). Larger, well-conducted reviews revealed that glucosamine hydrochloride had negligible effects on knee pain. In contrast, higher doses or higher-grade formulations of glucosamine sulfate (1,500 mg/day) and chondroitin (800 mg/day) had some statistically significant, though modest, benefits compared with placebo (178–181). For example, an industry-sponsored trial with 604 patients revealed that pharmaceutical-grade chondroitin sulfate was statistically superior to placebo and comparable to celecoxib in reducing pain and improving function. However, the clinical significance of these results was uncertain, as the degree of change in primary outcomes was minimal and similar across the chondroitin, celecoxib, and placebo groups. Other meta-analyses have indicated that glucosamine sulfate and chondroitin may slightly delay OA progression with long-term use (181–183). The placebo effect has been notable in studies involving these supplements, as exemplified by the Glucosamine/Chondroitin Intervention Trial (GAIT), where approximately 60% of participants experienced at least a 20% reduction in pain regardless of the treatment they received (184). In another trial, chondroitin sulfate plus glucosamine did not perform better than placebo in reducing global pain at 6 months, and the small sample size and dosing issues were limitations (185). Subgroup analyses revealed no difference in efficacy based on baseline pain severity or other factors (186). Similarly, vitamin D supplementation showed no benefit over placebo for pain relief or changes in cartilage volume in a large study (187, 188).

### Fish oil

A study comparing low-dose (0.45 g) to high-dose (4.5 g) fish oil (omega-3 fatty acids) revealed more significant improvements in pain and functional improvements with the lower dose over 2 years. Both doses had common gastrointestinal adverse events, such as upset and reflux. Fish oil has shown positive results in rheumatoid arthritis, likely due to its anti-inflammatory properties, but its effectiveness in treating OA remains unclear (187).

### Krill oil

Krill oil, known for its relatively high bioavailability of omega-3 fatty acids, has been tested for OA treatment. Two randomized trials with mild knee OA showed modest improvements in pain and stiffness with 2–4 g/day krill oil. However, a subsequent trial with moderate to severe knee OA revealed no significant benefits in pain relief or synovial inflammation compared with placebo, suggesting that krill oil may not be effective for more severe cases (189, 190).

### Phytoflavonoids

Phytoflavonoids, a group of natural compounds with anti-inflammatory properties, have shown potential in improving knee OA symptoms (191–193). However, specific phytoflavonoids, such as flavocoxid, are associated with serious adverse events, such as liver injury and hypersensitivity pneumonitis, making their use not recommended.

## Transcutaneous electrical nerve stimulation, acupuncture, local heat therapy, and cold therapy

### Transcutaneous electrical nerve stimulation

Transcutaneous electrical nerve stimulation (TENS) operates based on the gate-control theory, which posits that it modulates nociceptive signals to the brain through presynaptic inhibition in the dorsal horn of the spinal cord. Despite this theoretical basis, clinical trials have yielded mixed results. One study with 203 patients reported no additional benefits in pain relief or function from TENS, interferential currents, or shortwave diathermy compared with sham treatments combined with education and exercise programs (194). Another study involving 220 patients reported no significant difference between TENS and placebo TENS in WOMAC pain scores after 3 weeks (195). Moreover, evidence suggests a substantial placebo effect is associated with TENS (196).

### Acupuncture

A meta-analysis of randomized trials assessing acupuncture for knee osteoarthritis (OA) revealed that while acupuncture might offer some measurable benefits over sham acupuncture, these differences were not clinically significant (197). Similarly, a trial comparing six sessions of acupuncture, sham acupuncture, and no additional therapy in 352 adults reported no significant differences in pain scores among the three groups after 6 months (198). However, a larger multicenter trial involving 1,007 patients with chronic knee OA reported that after 10 sessions of acupuncture or sham acupuncture, success rates (defined as a 36% improvement in a standardized osteoarthritis index) were similar for both treatments and higher than those for conservative therapy alone [53% and 51% vs. 29%, respectively; (199)].

### Heat therapy

Local heat applications, such as heat packs or hot water bottles, can be a beneficial short-term strategy for pain relief in knee OA patients (200–202). A small cohort study demonstrated that combining local heat with routine management led to more significant improvements in pain and disability than routine management alone (202).

## Biological agents

Biological agents have shown significant effects in treating rheumatic disorders such as rheumatoid arthritis [RA; (203, 204)]. This success has spurred randomized controlled trials (RCTs) investigating biologic agents in osteoarthritis [OA; (205)]. These biotherapeutic strategies for OA aim to modulate or inhibit the effects of major cytokines, similar to the approach for RA treatment (206). The three main types of cytokine blockers used in OA target nerve growth factor (NGF), interleukin-1 (IL-1), and tumor necrosis factor-α (TNF-α), which are involved in OA pain pathways (207, 208). TNF-α, IL-1, and NGF can modulate pain through nociceptor sensitization, with NGF expression induced by the upregulation of IL-1 and TNF-α in OA (209, 210). Understanding the cytokine network in OA pathogenesis has strengthened the rationale for exploring whether this biotherapeutic approach can improve symptoms (Table 2).

**Table 2:** Comparative analysis of biologic agents in osteoarthritis—Key findings from recent studies.

**References**	**Type**	**Drug and target**	**Results**
Schnitzer et al. (223)	RCT	Tanezumab (NGF)	Statistically significant improvements in joint pain, physical function, and patient global assessment of osteoarthritis over 16 weeks; however, improvements were modest, and tanezumab-treated patients had more joint safety events and total joint replacements.
Chevalier et al. (219)	RCT	Anakinra (IL-1)	The mean improvements in the WOMAC score at week 4 were not statistically different between placebo and 50 mg (*P* = 0.67) or 150 mg (*P* = 0.77) of anakinra.
Lane et al. (215)	RCT	Tanezumab (NGF)	Average reductions in knee pain while walking ranged from 45% to 62% with tanezumab, compared with 22% with placebo (*P* < 0.001).
Nagashima et al. (224)	RCT	Tanezumab (NGF)	At week 8, tanezumab 25, 100, and 200 μg/kg improved various pain and function scores significantly compared to placebo.
Cohen et al. (213)	RCT	AMG108 100 (IL-1)	AMG108 showed numerically greater but statistically insignificant improvements in pain compared to placebo.
Brown et al. (212)	RCT	Tanezumab (NGF)	Tanezumab demonstrated superior analgesic efficacy in OA of the knee compared with placebo.
Sanga et al. (217)	RCT	Fulranumab (NGF)	Fulranumab significantly reduced average pain intensity score from baseline to week 12 compared with placebo (*P* ≤ 0.030).
Mayorga et al. (218)	RCT	Fulranumab (NGF)	Fulranumab monotherapy resulted in significantly better pain relief and function compared with oxycodone CR, but not against placebo.
Fleischmann et al. (220)	RCT	Lutikizumab (IL-1)	WOMAC pain score at week 16 improved significantly with 100 mg lutikizumab (*P* = 0.050) compared to placebo; no significant improvement with 25 mg or 200 mg doses.
Hochberg et al. (216)	RCT	Tanezumab (NGF)	Tanezumab 5 mg significantly improved pain and physical function but did not improve PtGA at week 16 compared to NSAIDs. Composite joint safety events were more prevalent with tanezumab 2.5 and 5 mg compared to NSAIDs (observation time-adjusted rate/1,000 patient-years: 38.3 and 71.5 vs. 14.8; *P* = 0.001 and *P* < 0.001, respectively).

However, controversy remains regarding the efficacy and safety of biologic agents in treating OA, with the literature presenting mixed outcomes of both success and failure (211, 212). Several studies have indicated that NGF inhibitors have effects on pain relief and functional improvement relative to placebo in OA, albeit with inconsistent safety performance (213, 214). For instance, clinical trials have demonstrated that tanezumab, an NGF inhibitor, resulted in significant reductions in pain and improvements in physical function compared to placebo (215). However, it was associated with a higher incidence of joint safety events and total joint replacements (216). Similarly, fulranumab, another NGF inhibitor, showed significant pain relief compared to placebo, but with variable results (217, 218).

In contrast, IL-1 inhibitors like anakinra and lutikizumab have shown limited success. Anakinra did not produce statistically significant improvements in pain scores compared to placebo (219), while lutikizumab had mixed results with significant improvements only at certain doses (220). TNF-α inhibitors, such as those investigated in some studies, were found to be ineffective for OA treatment in meta-analyses (221, 222).

Knee OA pain arises from inflammatory and mechanical mechanisms, necessitating tailored treatment strategies. Inflammatory pain benefits from anti-inflammatory agents like NSAIDs and corticosteroids, while mechanical pain is better addressed through interventions improving joint mechanics, such as hyaluronic acid injections and physical therapy. Many patients experience mixed pain, requiring a comprehensive approach that combines pharmacological treatments with supportive therapies such as exercise and weight management.

## Conclusions

Knee osteoarthritis (OA) is a common condition that significantly impacts quality of life and presents substantial health and economic challenges. Effective management requires a complex approach, which may include various treatment modalities.

Topical NSAIDs and capsaicin are effective initial therapies due to their safety and efficacy profiles. Oral analgesics, including acetaminophen and opioids, and intra-articular injections, such as corticosteroids and hyaluronic acid, provide varying degrees of relief but are limited by potential side effects.

Emerging evidence supports the potential benefits of mesenchymal stem cells for improving symptoms and joint function, though further research is necessary to confirm their long-term safety and efficacy. Nutritional supplements like curcumin and glucosamine-chondroitin offer modest benefits as adjuncts but lack robust evidence for primary therapy.

Non-pharmacological treatments, including TENS, acupuncture, and heat therapy, yield mixed results and should be tailored to individual patient responses. Biological agents targeting cytokines, such as TNF-α and IL-1, hold promise but require more extensive clinical validation.

Knee OA treatment should be personalized, balancing patient-specific factors and treatment preferences. An integrated approach combining pharmacological, non-pharmacological, and emerging biologic therapies offers the most effective pain relief and functional improvement strategy.
